# Comorbidity profiles of non-tuberculous mycobacteria infections in China: 12 years’ experience

**DOI:** 10.1128/spectrum.02018-25

**Published:** 2026-02-02

**Authors:** Chaohong Wang, Yiheng Shi, Bin Yang, Sibo Long, Weibing Lin, Dahong Su, Yan Zhao, Hao Li, Junhua Pan, Guirong Wang

**Affiliations:** 1 Department of Clinical Laboratory, Beijing Chest Hospital, Capital Medical University, Beijing Tuberculosis and Thoracic Tumor Institute 117550 , Beijing, China; 2 Department of Clinical Laboratory, Ningbo No.2 Hospital 74782 , Ningbo, Zhejiang, China; 3 College of Veterinary Medicine, China Agricultural University 34752, Beijing, China; 4 Beijing Chest Hospital, Capital Medical University, Beijing Tuberculosis and Thoracic Tumor Institute 117550 , Beijing, China; Inflammatix Inc., Sunnyvale, California, USA

**Keywords:** non-tuberculous mycobacteria, comorbidity, pulmonary disease, comorbidity

## Abstract

Nontuberculous mycobacteria (NTM) are of increasing public health concern. This study aimed to determine the most relevant comorbidities of NTM in Beijing, China, over a 12-year period. A retrospective analysis was performed on all 505 NTM patients diagnosed in Beijing Chest Hospital, Capital Medical University, from January 2010 to December 2021. Species identification, demographic characteristics, and comorbidities were analyzed using electronic medical records. The demographic characteristics and clinical comorbidity differences between NTM and tuberculosis (TB) patients were also analyzed. The number of patients infected with NTM in Beijing, China, has increased yearly. A total of 520 NTM were isolated from 505 inpatients, but only 465 were identified to the species level. *M. intracellulare* (43.44%, 202/465) and *M. abscessus* (128/465, 27.52%) predominated. NTM infection was associated with age, not gender. NTM infections were predominantly middle-aged (median 59 years, IQR 48.5–67) with a male-to-female ratio of 1:11. Notably, 86.1% of NTM disease had underlying conditions, most commonly malnutrition (43.0%), liver disease (31.1%), and bronchiectasis (25.0%), followed by previous TB (118/505, 23.37%), diabetes mellitus (63/505, 12.48%), hypertension (58/505, 11.49%), and pulmonary fungal infections (54/505, 10.69%). Gender-specific associations emerged: bronchiectasis was more prevalent in females (33.05% vs 17.67%, *P* < 0.001), while chronic obstructive pulmonary disease (COPD) predominated in males (9.77% vs 2.93%, *P*=0.002). Species-specific comorbidity patterns were identified: *M. intracellulare* was associated with malnutrition (50.50% vs 33.59%, *P* < 0.001) and diabetes (14.83% vs 4.69%, *P* < 0.001). Compared to TB, NTM cases showed stronger associations with structural lung diseases and immunosuppression. Clinical NTM infection cases increase year by year, with distinct species-specific comorbidity profiles. Our findings reveal that NTM disease is strongly associated with specific comorbidities, notably bronchiectasis, COPD, and immunological diseases. These conditions define a high-risk patient subgroup that requires targeted screening and proactive clinical management.

**IMPORTANCE:**

This study reveals a concerning rise in non-tuberculous mycobacteria (NTM) infections in Beijing, with *M. intracellulare* and *M. abscessus* as dominant species. Nearly 90% of patients had comorbidities like malnutrition, bronchiectasis, and liver disease, showing NTM's strong ties to weakened health. The work uncovers key differences from tuberculosis, including greater associations with lung damage and immunosuppression, plus gender-specific patterns (e.g., bronchiectasis in women). These findings highlight NTM as a growing threat requiring tailored diagnostic and treatment approaches, especially for vulnerable groups. The data provide crucial guidance for clinicians addressing this emerging challenge.

## INTRODUCTION

Nontuberculosis mycobacteria (NTM), a large group of mycobacteria distinct from *Mycobacterium tuberculosis complex* and *Mycobacterium leprosy* (1), are ubiquitous in natural environments such as soil and water (2, 3). Historically, NTM infections received little clinical attention because they are not transmitted from person to person and were often neglected as laboratory contamination (4, 5). However, the clinical significance of NTM has grown substantially, driven by advances in laboratory diagnostic technology, global population aging, and the increased population of immunocompromised hosts (6). Consequently, many recent reports concerned with morbidity and mortality of NTM have dramatically increased worldwide, particularly in countries with a high burden of tuberculosis (TB) (7), most of which are closely related to infectious diseases (8–10). Now recognized as one of the major emerging pathogens (11), NTM commonly causes pulmonary infections like *Mycobacterium tuberculosis*. The clinical symptoms and signs are often indistinguishable from those of TB, frequently leading to misdiagnosis as multidrug-resistant TB (12).

As a result, the diagnosis and treatment of NTM disease is challenging for clinicians (13). Host-pathogen interactions are pivotal in determining disease susceptibility, with factors such as age, sex, body mass index (BMI), and underlying comorbidities often affecting outcomes (14, 15).

In China, data on the demographic characteristics and clinical comorbidities of non-tuberculosis patients remain limited. A comprehensive analysis is especially needed for Beijing, the nation’s political and economic center. Hence, this retrospective study aimed to characterize the most relevant demographic characteristics and clinical comorbidities of NTM diseases in Beijing to provide a valuable reference for improving timely diagnosis and treatment.

## MATERIALS AND METHODS

### Species identification

The retrospective study included patients with NTM infections at Beijing Chest Hospital, Capital Medical University (Beijing, China), between January 2010 and December 2021 (a 12-year period). A total of 520 NTM specimens from 505 patients’ pulmonary and non-pulmonary sites were analyzed. The specimens obtained from ‘pulmonary sites’ included sputum, bronchial lavage fluid (BALF), and lung biopsy tissue. The ‘non-pulmonary’ site specimens included skin/soft tissue and lymph nodes. Direct smears were prepared from the above-separated specimens, stained with auramine, and observed by light-emitting diode microscopy.

After processing with NALC/NaOH and centrifugation, the supernatant of 500 μL was transferred into a 7 mL MGIT tube (Becton, Dickinson and Company, USA), and/or 100 μL was transferred onto LJ medium (Encode Medical Engineering Co., Ltd, China). LJ tubes were incubated at 37°C and examined weekly for growth for a maximum of 8 weeks, and MGIT tubes were cultured in the automated BACTEC MGIT 960 Mycobacteria culture system (BD), which usually reports positive results from two to four weeks and reports negative results after 42 days. All of the culture-positive isolates were primarily identified as *M. tuberculosis* complex (MTBC) by MPT64 antigen detection (Kaili Biotech kit, Hangzhou Innovation Biotech). Isolates that were initially identified as not MTBC by the MPT64 antigen testing were further identified to the species level using target DNA sequencing. We identified the isolates to species level by target DNA sequencing, including 16S rRNA, rpoB, hsp65, and the internal transcribed spacer region of the 16S-23S rRNA region (ITS) (16, 17). Genomic DNA was isolated from isolates by using the boiling method. There were 70 mycobacterial reference strains stored in the biobank in Beijing Chest Hospital (Beijing, China), which were obtained either from the American Type Culture Collection or the German Collection of Microorganisms. Multigene sequence similarity for the clinical isolates was determined in comparison with the reference sequences in our biobank or the multigene database using the basic local alignment search tool. Values above 99% sequence similarity for 16S rRNA and 97% similarity for hsp65, rpoB, and ITS genes were used for species distinction.

### Data collection and statistical analysis

The demographic information and clinical data of the patients were collected by electronic medical record system. The American Thoracic Society/Infectious Disease Society of America criteria were used to define cases of pulmonary or extrapulmonary site (6). For each NTM patient, we analyzed their age, gender, BMI, lab examination results, and comorbidities. Categorical variables were expressed as counts (percentages). Differences in frequencies were compared using the χ^2^ test or Fisher’s exact test. Continuous variables were expressed as median with 25–75th interquartile range (IQR), and the difference was assessed using the Mann-Whitney *U* test. We considered that *P* < 0.05 means statistical significance. SPSS version 25.0 and GraphPad Prism version 9.4.1 were utilized to conduct these statistical analyzes.

## RESULTS

### Prevalence of NTM and species distribution

**Fig 1:**
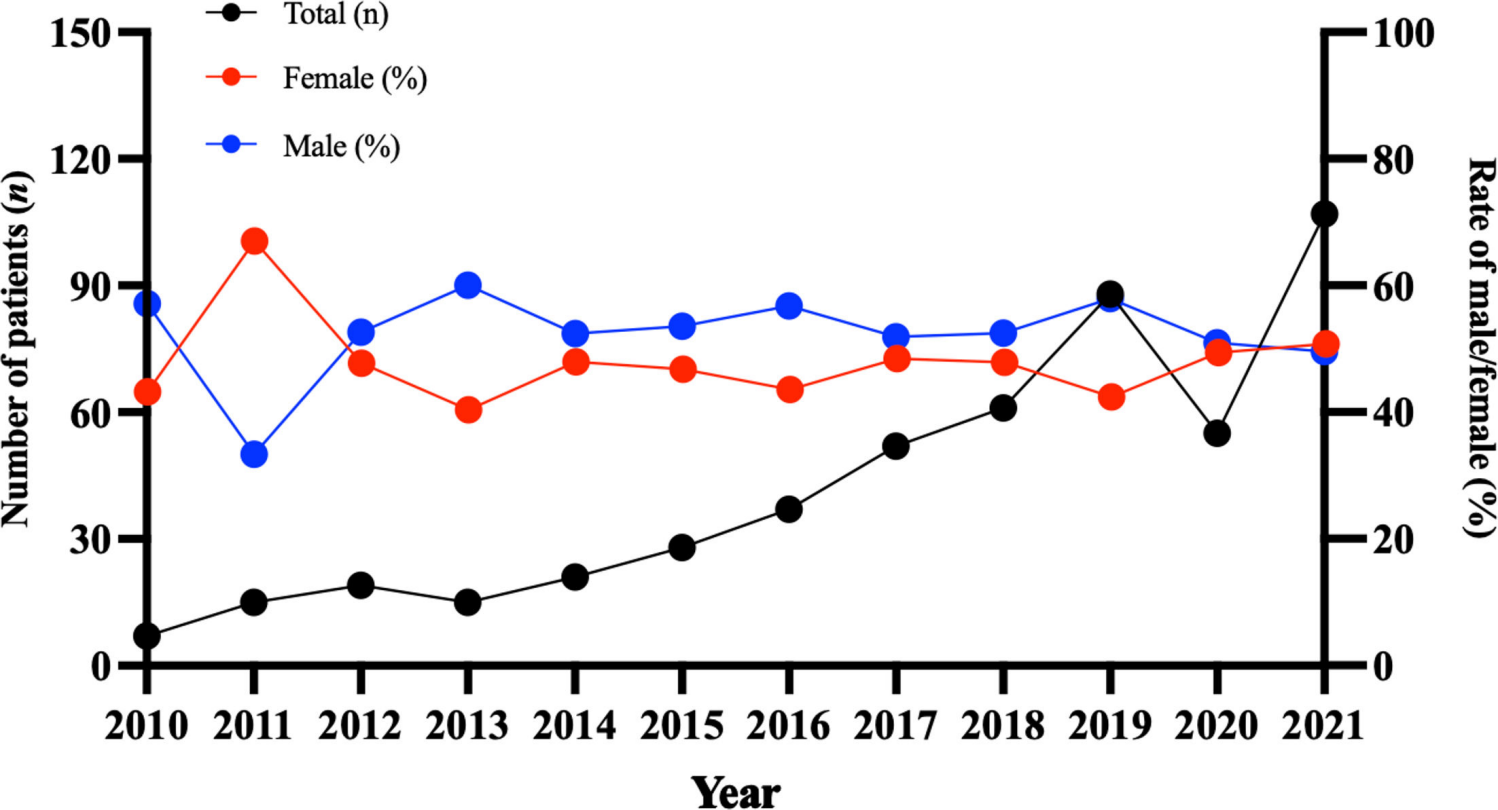
Number of diseases infected by NTM during the 12-year period (2010–2021).

Among 29,834 mycobacterial cases from January 2010 to December 2021 in Beijing Chest Hospital, 520 clinical NTM strains were isolated from 505 patients, of which 465 strains were identified as specific species. The annual number of NTM infections increased 15-fold from 7 cases (2011) to 107 cases (2021) (Fig. 1).

**Fig 2:**
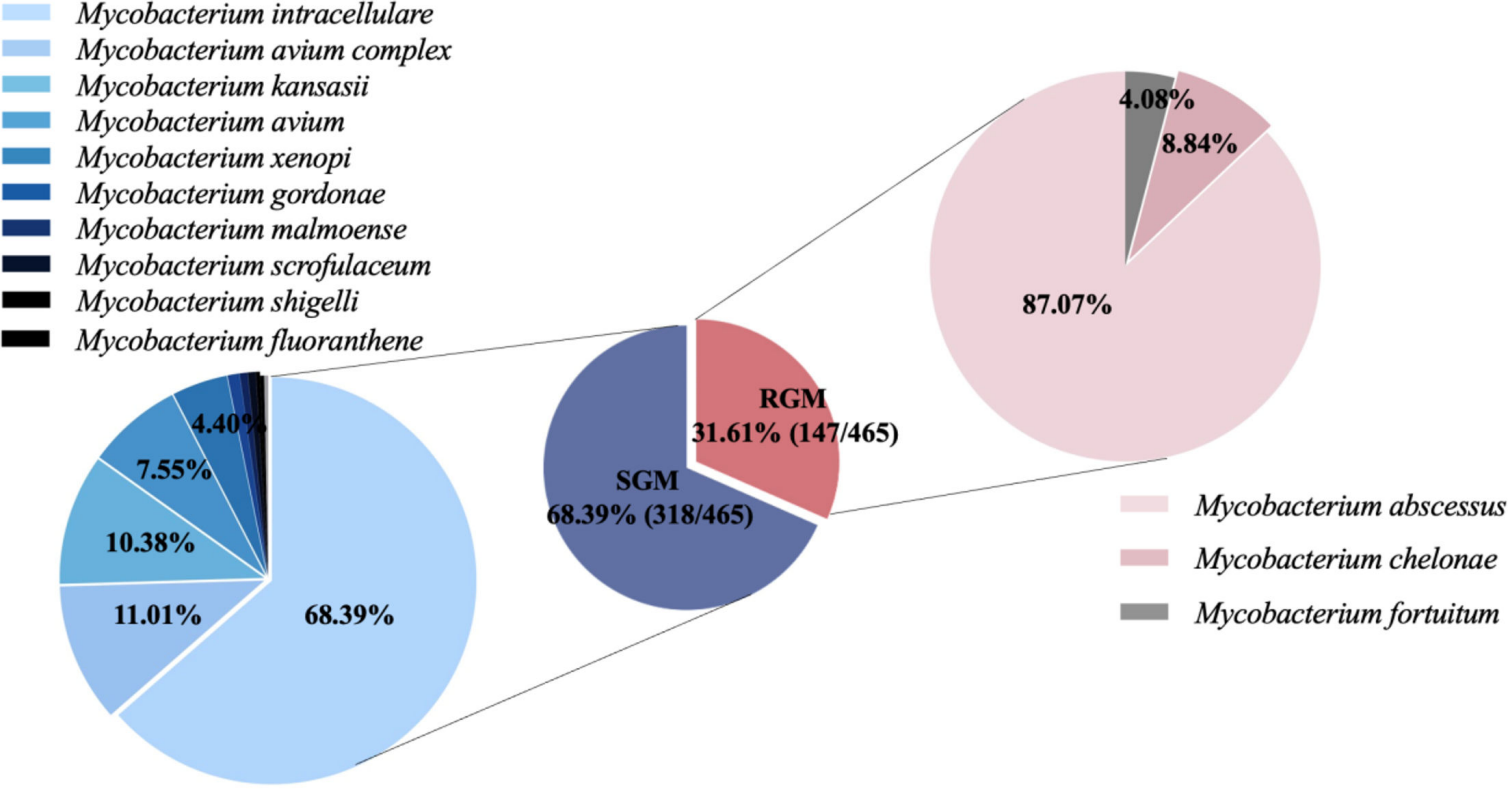
Frequency of species isolated from NTM patients during the study period.

During the 12-year study period, 14 patients were simultaneously infected with two or more NTM. The specimen types and detected species were shown in Tables 1 and 2. Among all analyzed NTM isolates, respiratory specimens were predominant (506/518, 97.68%), including sputum samples (407/518, 78.57%), BALF (71/518, 13.73%), and lung biopsy tissue (28/518, 5.41%). 13 patients (2.57%) had two or more specimens with a positive result. The isolation rate of slowly growing mycobacteria (SGM) is higher than that of rapidly growing mycobacteria (Fig. 2), and the top three prevalent species were *M. intracellulare* (202, 43.44%), *M. abscessus* (128, 27.52%) and *M. kansasii* (33, 7.10%) (Table 2).

**TABLE 1:** Sources of the specimens (*N* = 518)^*a*^

Specimen source	Sub-total, *N* (%)	Total, *N* (%)
Pulmonary		506 (97.68)
Sputum	407 (78.57)	
BALF	71 (13.73)	
Lung biopsy	28 (5.41)	
Extra-pulmonary		6 (1.16)
Skin/soft tissue	5 (0.97)	
Lymph node	1 (0.19)	
Unknown		6 (1.16)

^*a*^ BALF, bronchial lavage fluid.

**TABLE 2:** Frequency of NTM species isolated during the study period

NTM species	Sub-total, *N* (%)	Total, *N* (%)
Slowly Growing Mycobacteria		318 (68.39)
*Mycobacterium intracellulare*	202 (43.44)	
*Mycobacterium avium- intracellulare complex*	35 (7.53)	
*Mycobacterium kansasii*	33 (7.10)	
*Mycobacterium avium*	24 (5.16)	
*Mycobacterium xenopi*	14 (3.01%)	
*Mycobacterium gordonae*	3 (0.65%)	
*Mycobacterium malmoense*	2 (0.43%)	
*Mycobacterium scrofulaceum*	2 (0.43%)	
*Mycobacterium shigelli*	1 (0.22%)	
*Mycobacterium fluoranthene*	1 (0.22%)	
*Mycobacterium szulgai*	1 (0.22%)	
Rapidly Growing Mycobacteria		147 (31.61)
*Mycobacterium abscessus*	128 (27.52)	
*Mycobacterium chelonae*	13 (2.80)	
*Mycobacterium fortuitum*	6 (1.29%)	
Total		465 (100%)

### Demographic data and laboratory testing of the study patients

**Fig 3:**
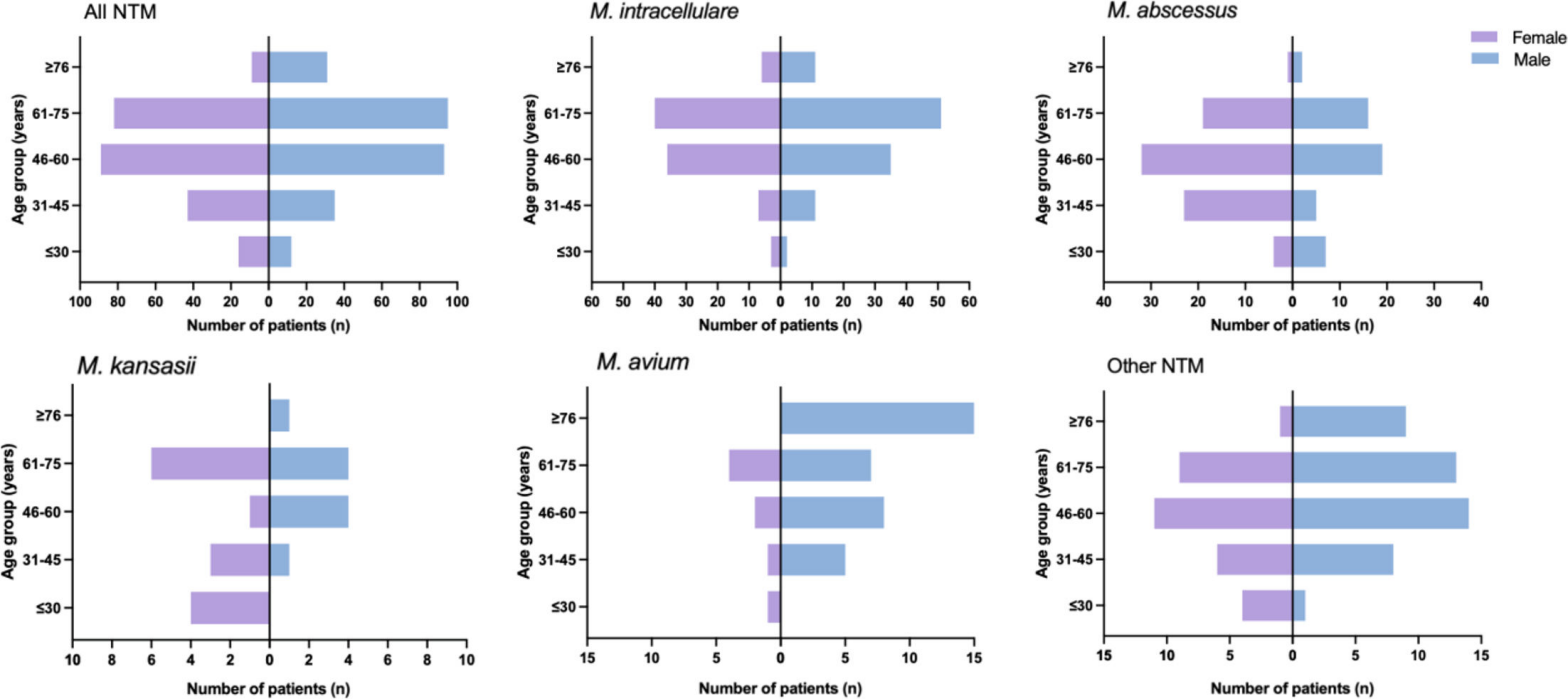
Age and sex distribution of patients with NTM during the study period.

According to the etiological results, 505 patients with NTM disease were included in this analysis. The median age of the cohort was 59 (48.5–67) with the majority of patients (71.09%, 359/505) aged between 46 and 75 years (Table 3 and Fig. 3). The cohort included 266 (52.67%, 266/505) male patients. At inclusion, most male and female patients had slim figures with a median BMI of 19.53 kg/m^2^ (17.28–21.97) (Table 3 and Fig. 3). By comparing the two groups with the highest isolation rate, 54.46% (110/202) of male patients were infected with *M. intracellulare* and only 38.28% (49/128) were infected with *M. abscessus*. From an age point of view, the most affected age category infected with *M. intracellulare* was 61–75 years old, while it was younger in patients infected with *M. abscessus*, mostly 46–60 years old.

**TABLE 3:** Demographics and clinical characteristics of the study population^*a*^

Characteristics	
Age (years): median (IQR)	59 (48.5–67)
Gender (male): *n* (%)	266 (52.67%)
BMI (kg/m^2^): median (IQR)	19.53 (17.28–21.97)
Previous history of PTB: *n* (%)	118 (23.37%)
Co-morbidities: *n* (%)	435 (86.14%)
Co-morbidities (pulmonary): *n* (%)	
Bronchiectasis	126 (24.95%)
Pulmonary fungal infection	54 (10.69%)
COPD	33 (6.53%)
Silicosis	9 (1.78%)
Pulmonary heart disease	9 (1.78%)
Pneumoconiosis	5 (0.99%)
Co-morbidities (non- pulmonary): *n* (%)	
Malnutrition	217 (42.97%)
Liver disease	157 (31.09%)
Diabetes mellitus	63 (12.48%)
Hypertension	58 (11.49%)
Coronary heart disease	45 (8.91%)
Immunological disease	45 (8.91%)
Malignancy	21 (4.16%)
Kidney disease	17 (3.37%)
Cerebrovascular disease	14 (2.77%)
Nervous system diseases	3 (0.59%)

^*a*^ COPD, Chronic Obstructive Pulmonary Disease.

Simultaneously, we recorded the erythrocyte sedimentation rate () of inpatients infected with NTM, 60.15% (246/409) of them had higher values.

### Comorbidities of NTM pulmonary disease

Among 505 NTM patients, 70 cases (13.86%) had no associated complications. Most patients had pre-existing lung disease or diseases related to nutritional deficiency. The most common comorbidity of NTM infection was malnutrition (217/505, 42.97%), liver disease (157/505, 31.09%), and bronchiectasis (126/505, 24.95%), followed by previous TB (118/505, 23.37%), diabetes mellitus (63/505, 12.48%), hypertension (58/505, 11.49%), and pulmonary fungal infection (54/505, 10.69%) (Table 3). In addition, we found that the incidence of bronchiectasis was higher in females (17.67%, 47/266 vs 33.05%, 79/239; χ^2^ = 15.914, *P* < 0.001), while Chronic Obstructive Pulmonary Disease (COPD) was found to be higher in males (9.77%, 26/266 vs 2.93%, 7/239; χ^2^ = 9.659, *P* = 0.002).

The comorbidities associated with the distribution of NTM species also vary. Patients with *M. intracellulare* were prone to malnutrition (102/202, 50.50%) and diabetes mellitus (30/202, 14.85%); on the contrary, patients with *M. abscessus were* more likely to be complicated with bronchiectasis (39/128, 30.47%) (Table 4).

**TABLE 4:** Relationship of comorbidities of patients with disease caused by the most common NTM^*a*^

	*M. intracellulare* (*N* = 202)	*M. abscessus* (*N* = 128)	*M. kansasii* (*N* = 33)	*M. avium* (*N* = 24)	*M. xenopi* (*N* = 14)	*M. chelonae* (*N* = 13)
Bronchiectasis	48 (23.76)	39 (30.47)	4 (12.12)	4 (16.67)	5 (35.71)	5 (38.46)
Pulmonary fungal infection	28 (13.86)	9 (7.03)	1 (3.03)	4 (16.67)	3 (21.43)	0 (0.00)
COPD	15 (6.44)	7 (5.47)	4 (12.12)	1 (4.17)	2 (14.29)	0 (0.00)
Silicosis	5 (2.48)	1 (0.78)	0 (0.00)	1 (4.17)	0 (0.00)	0 (0.00)
Pulmonary heart disease	3 (1.49)	1 (0.78)	0 (0.00)	1 (4.17)	0 (0.00)	0 (0.00)
Pneumoconiosis	2 (1.00)	0 (0.00)	1 (3.03)	1 (4.17)	0 (0.00)	0 (0.00)
Malnutrition	102 (50.50)*^b^	43 (33.59)*^b^	17 (51.52)	8 (33.33)	8 (57.14)	3 (23.08)
Liver disease	70 (34.65)	33 (25.78)	16 (48.48)	6 (25.00)	7 (50.00)	5 (38.46)
Diabetes mellitus	30 (14.85)*^b^	6 (4.69)*^b^	6 (18.18)	4 (16.67)	3 (21.43)	2 (15.38)
Hypertension	22 (10.89)	19 (14.84)	2 (6.06)	2 (8.33)	2 (14.29)	1 (7.69)
Coronary heart disease	17 (8.42)	4 (3.13)	5 (15.15)	2 (8.33)	1 (7.14)	1 (7.69)
Immunological disease	21 (10.40)	9 (7.03)	1 (3.03)	1 (4.17)	3 (21.43)	1 (7.69)
Malignancy	8 (3.96)	6 (4.69)	1 (3.03)	1 (4.17)	0 (0.00)	0 (0.00)
Kidney disease	5 (2.48)	6 (4.69)	0 (0.00)	3 (12.50)	1 (7.14)	0 (0.00)
Cerebrovascular disease	4 (1.98)	4 (3.13)	3 (9.09)	0 (0.00)	1 (7.14)	0 (0.00)
Nervous system diseases	2 (1.00)	1 (0.78)	0 (0.00)	0 (0.00)	0 (0.00)	0 (0.00)

^*a*^ COPD, Chronic Obstructive Pulmonary Disease.

^*b*^ * indicate* P *< 0.05 for comparison between *M. intracellulare* and *M. abscessus* by Chi-square test.

### Comparison analysis between NTM infections and TB

To further determine the risk factors for NTM disease, in addition to 505 patients with NTM infections, we also collected demographic characteristics and clinical comorbidities of 29,066 cases of TB, which were treated in our hospital in the same period. We noticed that there were statistically significant differences in age [59 (48.5–67) *vs*. 48 (28–63)], sex (1.11:1 *vs*. 1.78:1), and comorbidities (like bronchiectasis, pulmonary fungal infection, COPD, malnutrition, and immunological disease) between those infected with NTM and MTB (Table 5). The incidence of NTM was predominantly in the middle-aged and elderly, while TB is mainly in middle-aged males. Patients with NTM were worse in immunity and nutrition and were more likely to be complicated with lung diseases than patients with TB (Table 5). Hence, lung disease and immunocompromised status may play a vital part in the increased susceptibility to NTM infections.

**TABLE 5:** Comparison of demographics and clinical characteristics between patients with NTM and TB^*a*^

	NTM (*N* = 505)	TB (*N* = 29,066)	Z/χ^2^	*P* value	Effect size (95% CI)
Age (years): median (IQR)	59 (48.5-67)	48 (28-63)	−11.247	<0.000	11^*c*^
Gender (male): *n* (%)	266 (52.67%)	18,619 (64.06%)	27.876	<0.000	0.62 (0.52–0.75)^*d*^
Comorbidities: *n* (%)					
Bronchiectasis	126 (24.95%)	842 (2.91%)	762.459	<0.000	11.14 (8.93–13.83)^*d*^
Pulmonary fungal infection	54 (10.69%)	625 (1.47%)	161.471	<0.000	5.45 (3.99–7.33)^*d*^
COPD	33 (6.53%)	460 (1.59%)	74.251	<0.000	4.35 (2.92–6.28)^*d*^
Silicosis	9 (1.78%)	182 (0.63%)	–^*e*^	0.006^*b*^	2.88 (1.29–5.63)^*d*^
Pulmonary heart disease	9 (1.78%)	145 (0.50%)	–^*e*^	0.001^*b*^	3.62 (1.61–7.12)^*d*^
Pneumoconiosis	5 (0.99%)	130 (0.45%)	–^*e*^	0.082^*b*^	2.23 (0.71–5.36)^*d*^
Malnutrition	217 (42.97%)	8,850 (29.56%)	36.611	<0.000	1.72 (1.43–2.06)^*d*^
Liver disease	157 (31.09%)	8,789 (30.39%)	0.170	0.680	1.04 (0.86–1.26)^*d*^
Diabetes mellitus	63 (12.48%)	5,254 (18.17%)	10.558	0.001	0.65 (0.49–0.84)^*d*^
Hypertension	58 (11.49%)	4,320 (14.94%)	4.489	0.034	0.74 (0.55–0.98)^*d*^
Coronary heart disease	45 (8.91%)	1,719 (5.94%)	7.947	0.005	1.56 (1.12–2.13)^*d*^
Immunological disease	45 (8.91%)	599 (2.07%)	109.331	<0.000	4.65 (3.31–6.40)^*d*^
Malignancy	21 (4.16%)	1,567 (5.42%)	1.484	0.223	0.76 (0.47–1.18)^*d*^
Kidney disease	17 (3.37%)	1,239 (4.28%)	0.981	0.322	0.78 (0.45–1.27)^*d*^
Cerebrovascular disease	14 (2.77%)	902 (3.12%)	0.181	0.670	0.89 (0.48–1.52)^*d*^
Nervous system diseases	3 (0.59%)	40 (0.14%)	–^*e*^	0.037^*b*^	4.34 (0.86–13.69)^*d*^

^*a*^ COPD, Chronic Obstructive Pulmonary Disease.

^*b*^ Fisher's precision probability test.

^*c*^ Effect size for age is presented as the difference in medians.

^*d*^ Effect size for categorical variables is presented as the Odds Ratio (OR) with the TB group serving as the reference group.

^*e*^ –, not applicable.

## DISCUSSION

In this study, the amount of NTM is increasing year by year, which is consistent with the current trend of annual NTM isolates reported worldwide (18). We evaluated the demographic characteristics, distribution of strains, and clinical relevance of all NTM inpatients treated at Beijing Chest Hospital, Capital Medical University, from January 2010 to December 2021. There were a variety of pathogens; 13 kinds of NTM species were detected in total from different types of specimens (mainly from pulmonary), 10 SGM, and 3 RGM. The SGM were more commonly isolated than RGM (68.39% vs 31.61%), among which *M. intracellulare* was the predominant species, accounting for 43.44% of total disease-causing NTM, followed by 27.52% *M*. *abscessus*. These data were similar to the reports that *M. intracellulare* was the most common NTM species in China (19, 20), whereas they were different from Southeast Asian countries such as Japan and South Korea (21–23).

Published case reports and series showed that the age range of those most affected by NTM was over 60 years old, a trend linked to an aging population (24). Furthermore, slender women were more susceptible to NTM infection (25, 26). In our study, we also found that NTM infection was associated with age, not gender. The incidence of NTM infection is high in patients aged 46–75 years, with a median age of 59 (48.5–67), and the prevalence of pulmonary NTM disease was similar in male and female patients. However, both male and female patients had slim figures with BMI close to the lower end of the normal range (19.53 (17.28–21.97), 19.13 (17.29–21.46) vs 19.82 (17.15–22.41)).

Studies have shown that pre-existing chronic pulmonary diseases are described as major predisposing factors for the development of pulmonary NTM infection; structural lung diseases predispose people to pulmonary NTM infection (7, 27). Previous history of PTB, bronchiectasis, and COPD is frequently associated with NTM, as well as diseases such as pneumoconiosis, silicosis, and cystic fibrosis (28, 29). Weak immune systems and nutritional deficiency are also included in the risk factor list for NTM disease (27). Among the NTM population in this study, 86.14% of the patients were complicated with different kinds of comorbidities. Malnutrition (217/505, 42.97%), liver disease (157/505, 31.09%), and bronchiectasis (126/505, 24.95%) were the main comorbidities of NTM infection, followed by previous TB (118/505, 23.37%), diabetes mellitus (63/505, 12.48%), hypertension (58/505, 11.49%), and pulmonary fungal infection. Unfortunately, we have rarely observed patients with cystic fibrosis, which may be related to race. In addition, we did find something special; the incidence of bronchiectasis was higher in females (17.67%, 47/266; 33.05%, 79/239; χ^2^ = 15.914, *P* < 0.001). In contrast, COPD was found higher in males (9.77%, 26/266; 2.93%, 7/239; χ^2^ = 9.659, *P* = 0.002). These distinct features were consistent with the findings observed in other studies (30, 31). Another interesting finding is that the comorbidities associated with the distribution of NTM species also vary. Patients with *M. intracellulare* were prone to malnutrition and diabetes mellitus; on the contrary, patients with *M. abscessus* were more likely to be complicated with bronchiectasis. Compared with tuberculosis, NTM disease and other basic diseases of the lung are much inextricably linked. NTM infection is more common in patients with bronchiectasis, pulmonary fungal infection, and COPD. A high prevalence of NTM is associated with a high burden of pulmonary basic disease such as bronchiectasis, likely explained by pulmonary damage, thus increasing the risk of acquiring NTM. However, it is not clear whether NTM is the cause or consequence of bronchiectasis. In the meantime, we also found that the nutritional status and immune ability of patients with NTM disease were lower. Some patients had Sjögren’s syndrome and rheumatoid arthritis. Our results suggest that patients suffering from malnutrition may have abnormal laboratory test results. We recorded 60.15% (246/409) of NTM disease patients had hypoproteinemia or anemia and higher ESR; the latter can also reflect that these patients had chronic inflammation.

This study has several limitations. Firstly, the study population was from a single tertiary hospital. Secondly, there may be an underestimation in the prevalence of NTM, due to all the enrolled cases being HIV-uninfected, while HIV infection was a risk factor for NTM-PD. Finally, our analysis was based exclusively on patients with culture-confirmed NTM. While this approach ensures diagnostic accuracy, it introduces a potential selection bias, as our cohort does not capture patients with clinically suspected disease whose cultures remained negative. Therefore, the comorbidity profile we describe should be interpreted as representative of the culture-positive NTM population, which may not reflect the entire spectrum of NTM infection.

### Conclusion

Our work here underscores a fundamental point: NTM disease in our patients is intrinsically tied to specific underlying health conditions, setting it apart from tuberculosis. We found powerful links to bronchiectasis, COPD, and immunological diseases—associations so strong they should be considered defining features of the patient at risk. This isn't just an academic finding; it calls for a real shift in clinical practice. Instead of waiting for a classic presentation, clinicians should have a high index of suspicion for NTM in any patient with these comorbidities. For those with known bronchiectasis, in particular, proactive screening for NTM should be part of their routine care.
